# Incremental value of rare genetic variants for the prediction of multifactorial diseases

**DOI:** 10.1186/gm480

**Published:** 2013-08-20

**Authors:** Raluca Mihaescu, Michael J Pencina, Alvaro Alonso, Kathryn L Lunetta, Susan R Heckbert, Emelia J Benjamin, A Cecile JW Janssens

**Affiliations:** 1 Department of Epidemiology, Erasmus University Medical Center, Dr. Molewaterplein 50, Rotterdam, 3000 CA, The Netherlands; 2 Department of Biostatistics, Boston University, 801 Massachusetts Avenue, Boston, MA 02118, USA; 3 Harvard Clinical Research Institute, 930-W Commonwealth Avenue, Boston, MA 02215-1212, USA; 4 Division of Epidemiology and Community Health, School of Public Health, University of Minnesota, 1300 S. Second Street, Minneapolis, MN 55454-1015, USA; 5 The National Heart, Lung, and Blood Institute's Framingham Heart Study, 73 Mt. Wayte Avenue, Framingham, MA 01702-5827, USA; 6 Department of Epidemiology, University of Washington, Seattle, 1959 NE Pacific Street, Seattle, WA 98195-7236, USA; 7 Cardiology and Preventive Medicine Section, Boston University School of Medicine, Boston, 715 Albany Street, MA 02118, USA; 8 Department of Epidemiology, Boston University School of Public Health, Boston, 715 Albany Street, MA 02118, USA; 9 Emory University, Rollins School of Public Health, 1518 Clifton Road, Atlanta, GA 30322 USA

## Abstract

**Background:**

It is often assumed that rare genetic variants will improve available risk prediction scores. We aimed to estimate the added predictive ability of rare variants for risk prediction of common diseases in hypothetical scenarios.

**Methods:**

In simulated data, we constructed risk models with an area under the ROC curve (AUC) ranging between 0.50 and 0.95, to which we added a single variant representing the cumulative frequency and effect (odds ratio, OR) of multiple rare variants. The frequency of the rare variant ranged between 0.0001 and 0.01 and the OR between 2 and 10. We assessed the resulting AUC, increment in AUC, integrated discrimination improvement (IDI), net reclassification improvement (NRI(>0.01)) and categorical NRI. The analyses were illustrated by a simulation of atrial fibrillation risk prediction based on a published clinical risk model.

**Results:**

We observed minimal improvement in AUC with the addition of rare variants. All measures increased with the frequency and OR of the variant, but maximum increment in AUC remained below 0.05. Increment in AUC and NRI(>0.01) decreased with higher AUC of the baseline model, whereas IDI remained constant. In the atrial fibrillation example, the maximum increment in AUC was 0.02 for a variant with frequency = 0.01 and OR = 10. IDI and NRI showed at most minimal increase for variants with frequency greater than or equal to 0.005 and OR greater than or equal to 5.

**Conclusions:**

Since rare variants are present in only a minority of affected individuals, their predictive ability is generally low at the population level. To improve the predictive ability of clinical risk models for complex diseases, genetic variants must be common and have substantial effect on disease risk.

## Background

Genome-wide association studies (GWASs) have uncovered an incredible number of common susceptibility variants, but they explain only a small part of the heritability of complex diseases [1]. In search for the missing heritability, genetic research is investigating common variants with weak effects on disease risk, gene-gene interactions, structural variations and rare variants [2]. With the introduction of next generation sequencing, much effort is currently directed towards rare variants. Expected to have a predominant effect on protein structure, rare variants are more likely to be functional and to display strong effects on disease risk [3-6]. Sequencing of coding regions of the genome already has proved successful in identifying rare polymorphisms associated with common traits and complex diseases [7-10].

The predictive ability of rare variants and their potential to improve clinical risk models are uncertain for the population at large, as they are present in only a minority of the affected individuals. The predictive ability of rare variants in common diseases is understudied. Two methodological papers investigated the increment in area under the receiver operating characteristic curve (AUC) when rare variants were added to models based on common variants using simulated data [11,12]. They showed that the maximum increment in AUC was 0.06, but they did not provide the effect sizes of the rare variants, which makes it difficult to interpret the significance of their results. Additionally, AUC is considered an insensitive measure to detect potentially clinically important improvement in prediction [13-15]. Two new metrics were developed and rapidly gained popularity: the integrated discrimination improvement (IDI) and the net reclassification improvement (NRI) [16]. These metrics may be able to detect clinically significant improvement in prediction due to rare variants that the AUC fails to uncover.

We investigated the value of rare genetic variants for risk prediction of complex diseases. We examined the relation between the predictive ability of rare variants and their frequency, strength of effect (OR), and the predictive ability of the baseline risk model. We assessed the improvement in model performance by delta AUC (ΔAUC), IDI, and NRI. To this end, we simulated a large dataset and constructed risk models based on common variants for increasing values of the baseline AUC. In separate scenarios, we added rare genetic variants with varying odds ratios (OR) and frequencies. We further used hypothetical data that replicated the empirical populations used to derive a recently published clinical model for atrial fibrillation (AF) [17]. This common cardiac arrhythmia is associated with increased morbidity, mortality, and significant healthcare costs [18]. Numerous common genetic variants associated with atrial fibrillation risk have been identified [19-22] and rare genetic variants are expected to improve the detection of at-risk individuals [23,24].

## Methods

### Simulation of data

First, we used a simulation procedure to investigate the effect of the predictive accuracy of the baseline model on the discrimination of the model updated with rare variants. The modeling procedure has been described in detail by Janssens et al. [25]. In short, this procedure creates a dataset of genotypes for a hypothetical population. Genotypes, coded as 0, 1, or 2 based on the number of risk alleles, are assigned in such a way that the allele frequencies of the genetic variants match specified values and are in Hardy-Weinberg equilibrium. By changing the number, frequency, and ORs of simulated variants we created baseline models with an AUC ranging between 0.50 and 0.95. We added rare genetic variants to the simulated dataset of common variants. Rare variants were simulated as a single variant representing multiple rarer variants. That is, for example, 20 independent rare variants each with a frequency of 1 in 2,000 individuals can collectively be viewed as a single variant with a frequency of 0.01. The variant was coded as 1 or 0 if the individual carried any or none of the risk alleles. We simulated rare variants with a frequency of 0.0001, 0.001, 0.005, or 0.01, and an OR of 2, 5, or 10. We used arbitrary values for the parameters of the rare genes, but based our choice on the literature [3,26-28].

To compare the added value of rare and common variants for risk prediction, we also simulated 10 to 100 common variants each with a risk allele frequency of 0.05 or 0.30 and an OR of 1.10 or 1.05. We have used these parameters because most of the approximately 400 single nucleotide polymorphisms (SNPs) discovered in 100 recent GWAS had an OR of approximately 1.10 and future GWAS efforts are expected to uncover SNPs with even lower effect sizes [28]. Disease risk was 4% as in the AF example, or 10% to examine the impact of higher disease risks on the measures of predictive ability. In the simulations, disease risk can be interpreted as a disease incidence, for example, a disease incidence over 5 years. For both the main simulations and the AF example, population size was 200,000 for scenarios in which rare variants were added and 20,000 for scenarios in which common variants were added. Predicted risks for each individual were obtained from logistic regression analyses, were calculated in the range 0 to 1 and were rounded to two decimal points.

### Simulation study of atrial fibrillation

#### Background for choosing the example of atrial fibrillation

Complex diseases can be multifactorial, that is, caused by an intricate effect of multiple environmental and genetic risk factors, but can also include monogenic forms. One such example is AF, which consist of a rare familial form that is a monogenetic disease and a common non-familial form [29]. Targeted use of prevention strategies is warranted to reduce the burden of AF, which requires accurate detection of individuals at high risk. Algorithms for detection of individuals at risk, based on routinely collected clinical risk factors, have already been developed and validated in various populations [17,30,31]. The predictive accuracy of these clinical scores leaves ample opportunity for improvement, and so fuels the research for finding new biomarkers, including genetic variants [23,32,33]. Several susceptibility variants for AF have been found [19-21] but their combined predictive ability is low as they explain only a fraction of the heritability [1]. While sequencing efforts are ongoing for AF, research focused on the potential use of rare variants for risk prediction of AF becomes very relevant [23,24]. We assessed the incremental value of rare genetic variants over an existing clinical risk score for AF.

#### Methods for constructing the dataset of clinical and genetic risk factors

To assess the value of rare variants for AF risk prediction we simulated a hypothetical population that reflected the characteristics of the community-based cohort in which the clinical risk score was developed (that is, the combination of Atherosclerosis Risk in Communities Study, Cardiovascular Health Study and Framingham Heart Study; please see Additional file for details on study design) [34-36]. We simulated the distribution of clinical and genetic risk factors separately in individuals with and without the outcome by random sampling from a multivariate normal distribution. To derive categorical clinical variables and genetic variants, we transformed the corresponding continuous variables into categorical variables. We simulated clinical variables to be correlated, as observed in the empirical population (see Additional file, Table S3). We assumed that genetic variants were uncorrelated with one another and were uncorrelated with clinical risk variables. Detailed information about the simulation strategy is provided in the Supplementary Methods (see Additional file).

#### Description of the clinical model

Variables included in the clinical risk score were: age, race, smoking status, weight, height, systolic blood pressure (SBP), diastolic blood pressure (DBP), diabetes, medication for hypertension, history of congestive heart failure, and history of myocardial infarction (see Additional file, Table S1 for the distribution of clinical variables). In the empirical dataset, that is, the combination of Atherosclerosis Risk in Communities Study, Cardiovascular Health Study and Framingham Heart Study, the outcome was defined as AF during 5 years of follow-up. Individuals were free of AF at the beginning of follow-up. The disease incidence was 4%. Simulations accurately replicated the empirical data (see Additional file, Table S4).

#### Description of genetic variables

We used the same parameters for the rare variants as in the simulation scenarios where we varied the baseline AUC. To estimate the added value of recently identified susceptibility single nucleotide polymorphisms (SNPs) for AF, we added to the clinical variables 10 genetic variants with the same frequency and OR as the top 10 (that is, in terms of *P *value) uncorrelated SNPs from a recent meta-analysis performed in the CHARGE AF consortium [22] (see Additional file, Table S2).

### Metrics

We assessed discrimination of the baseline, genetic, and combined models; improvement in discrimination; and clinical usefulness of updating the baseline model with genetic variants. We used AUC as a global measure of discrimination. AUC indicates the degree to which the predicted risks can discriminate between individuals who will and will not develop the disease. AUC generally ranges from 0.50 (equal to tossing a coin) to 1.00 (perfect discrimination). We used the increment in AUC (ΔAUC), IDI, and continuous NRI as measures of global improvement in discrimination. IDI was calculated as the difference in mean predicted probabilities between cases and controls between the two models [16]. NRI is an overall measure of correct reclassification of cases to higher risk categories and of controls to lower risk categories [16]. The continuous NRI [NRI(>0)] does not use categories but takes into account any increase or decrease in predicted risk produced by the model update [37]. Since we rounded risks to 0.01 (that is, 1%) the NRI without categories used here is denoted as NRI(>0.01). In other words the minimal change in risk is 0.01. We used categorical NRI to assess clinical usefulness. Clinical usefulness concerns the reclassification of individuals in risk categories that leads to changes in preventive or therapeutic interventions. We defined three risk categories by using the risk cutoffs of 2.5% and 5%, similar to those used the evaluation of the clinical risk score for AF [17]. We also report the NRI in cases and controls separately, as this may provide additional insight into the impact of model update [16,37]. For scenarios with various baseline AUC, we calculated ΔAUC, NRI(>0.01), and IDI. For scenarios with the clinical risk score we calculated ΔAUC, IDI, NRI(>0.01), and categorical NRI.

Reported measures are median results from 200 simulations unless stated otherwise. All analyses were performed using the R programming language, version 2.11.1 [38].

## Results

### Simulation analyses

**Figure 1:**
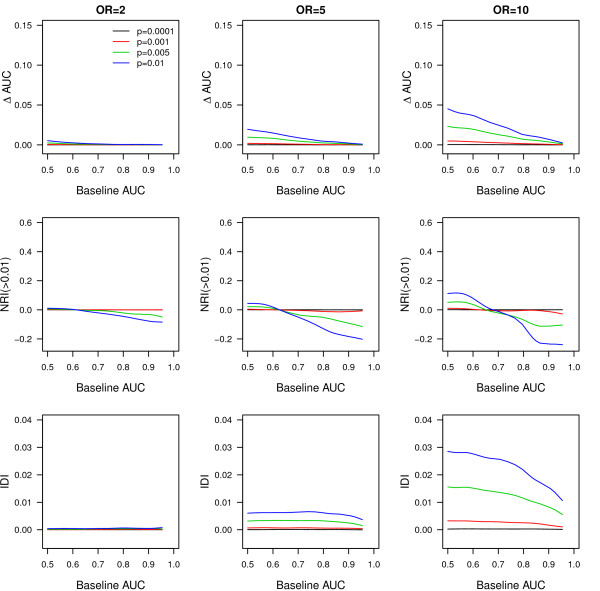
**Change in AUC, NRI(>0.01) and IDI per different values of the baseline AUC when rare genetic variants were added to prediction baseline model**. ΔAUC, change in AUC between the model with and without the rare genetic variants; AUC, area under the receiver operating characteristic curve; IDI, integrated discrimination improvement; n, number of common variants added; NRI, net reclassification improvement; OR, odds ratio; p, frequency of the risk allele; AUC 1, AUC of the baseline model. Disease risk is 4%. Population size is 200,000. Results are median values from 10 simulations.

Figure 1 shows that, for a disease risk of 4%, the median AUC and NRI(>0.01) only improved when variants were not very rare and had higher ORs, and only when baseline AUC values were in the lower range. Across higher baseline AUC values, the median NRI(>0.01) became negative, suggesting that rare variants produced more wrong than correct risk reclassifications. The median IDI was close to zero for very rare variants and minimally increased with higher frequency and OR of the rare variants. The median IDI was constant across most baseline AUC values. When disease risk was higher (that is, 10%), most performance measures slightly increased compared to the scenarios with lower disease risk (see Additional file, Figure S1). For rare variants with OR = 10 and frequency ≥0.005, the median increment in AUC varied between 0.01 and 0.05 depending on the value of the baseline AUC. The median NRI(>0.01) varied between 0.18 and 0.55 and, in contrast to the scenario with the lower disease risk, increased with a higher baseline AUC.

**Figure 2:**
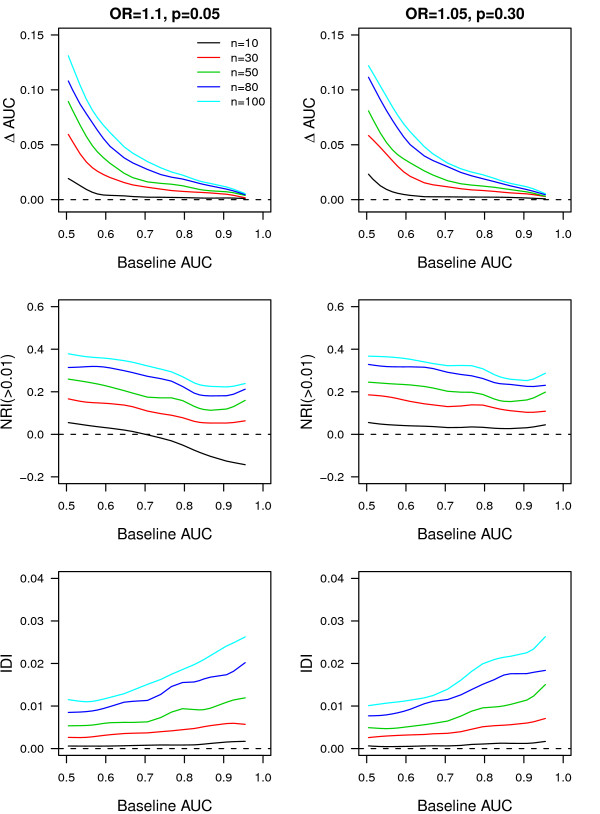
**Change in AUC, NRI(>0.01) and IDI per different values of the baseline AUC when common genetic variants were added to prediction baseline model**. ΔAUC, change in AUC between the model with and without the common genetic variants; AUC, area under the receiver operating characteristic curve; IDI, integrated discrimination improvement; n, number of common variants added; NRI, net reclassification improvement; OR, odds ratio; p, frequency of the risk allele; AUC 1, AUC of the baseline model. Disease risk is 4%. Population size is 20,000. Results are median values from 10 simulations.

As a comparison, we investigated the addition of 10 to 100 common variants, each with a frequency of 0.05 and an OR of 1.10 or a frequency of 0.30 and an OR of 1.05. We found a higher increase in AUC compared to the addition of rare variants (see Figure 2). NRI(>0.01) was mostly positive and increased with the number of variants added. In contrast, IDI was minimal even with the addition of 100 variants. Surprisingly, although the increment in AUC was higher, the IDI was in some instances lower for common variants compared to rare variants. As such, across low baseline AUC values rare variants with OR = 10 and frequency ≥0.005 showed higher IDI than 100 common variants. This trend was seen also when disease risk was higher (that is, 10%) (see Additional file, Figure S2). Furthermore, rare variants with OR = 10 and frequency ≥0.005 showed also higher NRI(>0.01) across higher baseline AUC values compared to 100 common variants (see Additional file, Figure S2).

**Figure 3:**
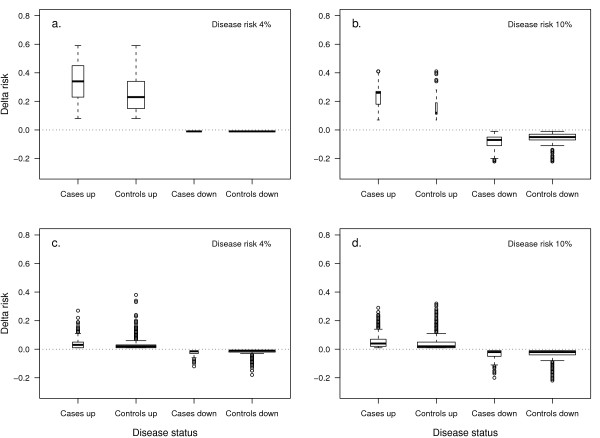
**Change in absolute risk at model update with rare and common genetic variants**. On the × axis is shown the correct reclassification of cases and controls (that is, Cases up; Controls down) and incorrect reclassification (that is, Cases down; Controls up) when rare variants with a cumulative OR of 10 and frequency of 0.01 (Figures 3a, b) or 100 common variants each with a OR of 1.05 and a frequency of 0.30 (Figures 3c, d) are added to a baseline model with an AUC = 0.70. The bold line shows the median, the boxes indicate the interquartile ranges (range, 25-75%), and the whiskers present 1.5 times the interquartile range. Box widths are proportional to the square-root of the number of individuals in the groups. Disease risk is 4% in Figures 3a and c, and 10% in Figures 3b and d. The plot is obtained from one simulation using 200,000 individuals for Figures 3a and b, and 20,000 individuals for Figures 3c and d.

To investigate the added value of rare and common variants at the individual level, we additionally assessed the magnitude of change in absolute risk at reclassification. Having a rare variant substantially increased the risk in <1% of both cases and controls when disease risk was 4% (median increase in absolute risk: 0.35 in cases and 0.24 in controls; see Figure 3a). When disease risk was 10% the risk in 10% of cases largely increased while the risk in <1% of controls increased only marginally (median increase in absolute risk: 0.78 in cases and 0.02 in controls; see Figure 3b). The median decrease was negligible in both cases and controls that did not carry the risk variant (that is, -0.01). In contrast, when 100 common variants were added to the model and the disease risk was 4%, the risk minimally increased or decreased in individuals that were reclassified to higher or lower risk categories (median increase in absolute risk: 0.03 in cases and 0.02 in controls, median decrease: -0.02 and -0.01; see Figure 3c). When common variants were added, about a half of the cases and controls moved in the right direction while around one-quarter moved in the wrong direction. Similar results were observed when disease risk was 10% (see Figure 3d). Besides the individuals that carried the risk variant, an increase in risk was observed also in some individuals that did not carry a rare variant. This risk increase was minimal and was due to a difference in beta coefficients between the two regression models (data not shown).

### Clinical example: genetic prediction of atrial fibrillation

The median AUC of the clinical model was 0.76 (95% confidence interval, 0.75 to 0.78). Rare variants improved the AUC of the clinical model only when they were more frequent and had very high OR (see Table 1). Adding a rare variant with a frequency of 0.01 increased AUC by 0.02 when OR was 10, but did not improve AUC when OR was 2. IDI and NRI were zero for very rare variants, that is, when frequency was 0.0001 or 0.001. IDI minimally increased with a higher frequency and OR of the rare variant (see Table 1). A variant with frequency of 0.01 and an OR of 10 yielded an IDI of 0.03. NRI(>0.01) was negative in most scenarios. The higher the frequency and OR of the rare variant, the larger the negative value of NRI(>0.01). In contrast to the NRI(>0.01), categorical NRI and NRI in controls, but not in cases, were positive and minimally increased with the frequency and OR of the rare variants. Adding 10 variants with empirical ORs and frequencies showed a minimal improvement in all model performance measures.

**Table 1:** Performance of genetic and combined (clinical and genetic) risk models for AF using rare and common variants.

OR	Frequency	Variants (*n*)	AUC			IDI	NRI(>0.01)		NRI categorical	
						
			Genetic	Combined	Δ			Total	Cases	Controls
**Rare variants**										

**2**	**0.0001**	1	0.50	0.76	0	0	0	0	0	0
	**0.001**	1	0.50	0.76	0	0	0	0	0	0
	**0.005**	1	0.50	0.76	0	0	-0.01	0	0	0
	**0.01**	1	0.51	0.76	0	0	-0.03	0	0	0

**5**	**0.0001**	1	0.50	0.76	0	0	0	0	0	0
	**0.001**	1	0.50	0.76	0	0	-0.01	0	0	0
	**0.005**	1	0.51	0.76	0	0	-0.05	0.01	0.01	0
	**0.01**	1	0.52	0.77	0.01	0.01	-0.10	0.02	-0.01	0.03

**10**	**0.0001**	1	0.50	0.76	0	0	0	0	0	0
	**0.001**	1	0.50	0.76	0	0	-0.03	0.00	0	0.01
	**0.005**	1	0.52	0.77	0.01	0.02	-0.11	0.02	-0.01	0.03
	**0.01**	1	0.54	0.78	0.02	0.03	-0.13	0.04	-0.02	0.06

**Common variants**										

**1.14-1.45^a^**	0.03-0.84*	10	0.59	0.77	0.01	0.01	0.20	0.04	0.01	0.04

^a^Using parameters from the top 10 (that is, in terms of *P *value) uncorrelated SNPs in the CHARGE AF meta-analysis; in the table are listed the range of OR and allele frequency [22]. Variables included in the clinical risk score were: age, weight, height, systolic blood pressure (SBP), diastolic blood pressure (DBP), diabetes, medication for hypertension, history of congestive heart failure, history of myocardial infarction, smoking status, and race. Disease risk is 4% and population size is 200,000 for rare variants scenarios and 20,000 for common variants scenarios. Results are median values from 200 simulations.

AUC, area under the receiver operating characteristic curve; ΔAUC, change in AUC between the model with and without genetic variants; IDI, integrated discrimination improvement; NRI, net reclassification improvement (cutoffs 2.5% and 5%); OR, odds ratio.

## Discussion

Using a hypothetical population, we have shown that rare variants only minimally improved AUC and did not yield clinically relevant positive NRI(>0.01) and IDI when disease risk was low. Rare variants produced larger increments in AUC when the baseline model had lower AUC, but in these scenarios NRI(>0.01) and IDI remained close to zero. Addition of rare variants to the baseline model largely increased predicted risks for the few individuals carrying the risk variant, whereas predicted risks were only slightly decreased for those who did not carry the variant. For a higher disease risk, rare variants with strong effects showed improvement in AUC across a wider range of baseline AUC values and significant positive NRI(>0.01) and IDI. Addition of rare variants to the baseline model largely increased predicted risks only in cases, as expected.

Before addressing the implications of these results for future research, we discuss several methodological aspects of our study that might have impacted the results. First, we modeled rare variants as a single variant. This is a common procedure used to investigate the association of multiple extremely rare variants with disease risk and does not affect the results presented here [9,39]. Second, we assumed that each genetic variant was uncorrelated with other variants and clinical risk factors. While linkage equilibrium between rare variants is a very realistic assumption, rare variants may be in linkage disequilibrium with common variants. In fact, it has been suggested that common variants may share a haplotype with the true rare causal variants [6]. Third, genetic variants may be associated with intermediate risk factors for disease, which are often the variables included in traditional clinical risk scores. Such correlations would likely decrease the impact of the variants and hence show less improvement in performance than reported in this paper.

We have shown that, from a population perspective, rare variants are only useful for risk prediction of complex diseases when they have strong effects on disease risk, when they are not too rare and when the risk of disease is high. Figure 1 shows that when disease risk was 4% the addition of rare variants resulted in an improvement in AUC only when the baseline AUC was low. As shown, this trend was more pronounced when rare variants had higher OR. NRI(>0.01) showed a minimal added value of rare variants only when baseline AUC was lower (≤0.70) and variants had very strong effects (OR = 10). When baseline AUC was ≥0.80, the NRI(>0.01) indicated that overall more wrong reclassifications of risk were done by addition of rare variants. To summarize, rare variants only improved discrimination when baseline AUC values were low, but even then the improvement was minimal. Only when the OR of the rare variant was very large, its frequency higher and disease risk high, were the AUC and NRI(>0.01) improved across a wider range of baseline AUC. If the expected effect sizes might be smaller than previously thought, the predictive ability of rare variants will be even lower than our results indicate. The NRI(>0.01) and IDI values were higher than those observed with the addition of 100 common variants with a frequency and OR as used in this study. Thus, despite a lower improvement in AUC, rare variants may result in larger improvements in NRI(>0.01) and IDI compared to common variants. This apparent discrepancy in observations is explained by the fact that AUC only considers the rank in predicted risks, not actual values, whereas NRI and IDI do depend on actual magnitude of changes in predicted risks before and after updating the model. Rare variants with strong effect by definition have a substantial impact on disease risk, albeit for a small group of individuals.

Interestingly, the degree of precision had a large impact on the global improvement of discrimination as measured by continuous NRI. The discrepancy in results was most striking for the rare variants. As such, we observed a NRI(>0) of 0.17 when a rare variant with OR of 10 and frequency of 0.01 was added to the clinical AF model and predicted risks were not rounded, compared to an NRI(>0.01) of -0.13 when risks were rounded to two decimal points. This is likely explained by the fact that, by definition, most individuals did not carry the rare risk variant and this resulted in a very small decrease in risk for most individuals, a change that was not captured when risks were rounded. In contrast, the difference in AUC and categorical NRI between non-rounded and rounded risks was minimal, that is, at most 0.01 in a few scenarios from the AF example. This raises the question what is the amount of precision to be reported for risk predictions and what is the most appropriate continuous NRI measure.

## Conclusions

In conclusion, we have shown that addition of rare variants to baseline risk models that include clinical or genetic risk factors resulted in model improvement only when the rare variants had strong effects on disease risk. This improvement was larger with a higher disease risk because the odds ratios lead to different likelihood ratios when the disease is more common. We have also shown that rare variants largely increased the risk in some individuals, while most individuals were reclassified to a slightly decreased risk. Very rare variants, by definition, occur in only few individuals that ultimately develop the disease and therefore have poor sensitivity and a limited predictive ability. This means that most individuals will either not be reclassified into another risk category or will be reclassified on the basis of clinically irrelevant changes in predicted risks. Counterintuitively, most individuals who will develop the disease will see their risk slightly decreased after testing for rare variants. Although this decrease in disease risk is minimal, individuals with predicted risks just above the threshold may be moved to a lower risk category. In the case of AF, a disease associated with stroke and increased mortality [40], this would mean that many individuals would not benefit from the potentially lifesaving preventive measures.

While rare variants are unlikely to improve the prediction of common diseases in the population, they do have substantial impact on disease risk in carriers of the rare variants. When rare variants have very strong effects on disease risk, they are probably more aggregated within certain families and resemble a Mendelian transmission. It would be of high interest to compare family history information [41] with tests including rare variants and, further, to investigate if such variants can be more predictive in families with positive family history. Apart from such exceptions, it should be anticipated that the study of rare variants will have its largest contribution in advancing our understanding of disease pathophysiology.

## List of abbreviations

AF: atrial fibrillation; AUC: area under the ROC curve; ΔAUC: increment in AUC; DBP: diastolic blood pressure; GWAS: genome-wide association study; IDI: integrated discrimination improvement; NRI: net reclassification improvement; OR: odds ratio; SBP: systolic blood pressure; SNPs: single nucleotide polymorphisms.

## Competing interests

The authors declare that they have no competing interests.

## Authors' contributions

RM, MJP, and ACJWJ conceived the study and drafted the manuscript. RM performed the statistical analysis. AA, KLL, SRH, and EJB participated in the design and helped to draft the manuscript. All authors read and approved the final manuscript.

## Description of additional data files

The following additional data are available with the online version of this paper. The Additional file includes information on study design and baseline characteristics in Atherosclerosis Risk in Communities Study, Cardiovascular Health Study and Framingham Heart Study and describes the top 10 independent SNPs, change in AUC, NRI(>0.01), and IDI per different values of the baseline AUC when rare or common genetic variants are added to prediction baseline model and additional information on the methods.
